# Scoping review of biological and psychosocial pathways that lead from childhood adversity to early-onset substance use

**DOI:** 10.3389/fpsyt.2025.1612494

**Published:** 2025-06-18

**Authors:** Maite Ramírez, Asier Ugedo, Lourdes Fañanás, Guilermo Cano-Escalera, Pilar A. Saiz, Iñaki Zorrilla, Ana González-Pinto

**Affiliations:** ^1^ Department of Psychiatry, IIS BIOBIZKAIA, University of the Basque Country (EHU), CIBERSAM, Hospital Universitario Galdakao-Usansolo, Galdakao, Spain; ^2^ Department of Psychiatry, Hospital Universitario Galdakao-Usansolo, Galdakao, Spain; ^3^ Department of Evolutionary Biology, Ecology and Environmental Sciences, Faculty of Biology, Biomedicine Institute of the University of Barcelona (IBUB), CIBERSAM, University of Barcelona, Barcelona, Spain; ^4^ BIOARABA. Psychiatry Unit, Vitoria-Gasteiz, Spain; ^5^ Department of Psychiatry, University of Oviedo, Oviedo, Spain; ^6^ Department of Psychiatry, BIOARABA, University of the Basque Country (EHU), CIBERSAM, Hospital Universitario de Alava, Vitoria-Gasteiz, Spain

**Keywords:** scoping review, adolescents, early-onset, substance use, childhood, adversity, maltreatment

## Abstract

**Background and objectives:**

Substance use in children and adolescents exposed to childhood adversity is a recognized risk factor for adverse outcomes in mental and physical health. However, few studies focus on the specific mechanisms that lead to it, assuming they are similar to those in adults. The purpose of this review is to explore the existing literature regarding etiological pathways between environmental adversities in childhood and early-onset substance use.

**Methods:**

a scoping review was conducted following PRISMA-ScR criteria, as the evidence is complex, heterogeneous, and relatively underexplored. Two independent reviewers searched Medline, Embase, PsycInfo, Web of Science, and grey literature individually for review studies on biological and psychosocial pathways that lead from childhood adversity to early onset substance. Only outcomes that applied to children and adolescents under 18 years were recorded.

**Results:**

Pathways that lead from childhood adversity to early-onset substance use appear to be multifactorial and non-linear. Stress induces changes in vulnerable neural circuits, affecting emotion regulation, decision-making, and intrapersonal and interpersonal functioning. These changes and additional drug-induced effects on the developing brain provoke a cascade of events that increase the risk of heavy and uncontrollable use.

**Conclusion:**

Developmental stage-specific factors may influence substance use in adolescents exposed to childhood adversity. Identifying mediators in this high-risk population is crucial to implementing efficacious preventive strategies.

## Introduction

The etiology of mental disorders has been usefully conceptualized as an interaction between predisposing and triggering factors (1). Regarding substance use, a potent risk factor is the experience of traumatic and other chronic or severely stressful events in childhood (2, 3). However, a linear progression along this pathway is rarely observed, as individuals show considerable variability in the likelihood of early experimental use and significant fluctuations in usage patterns, escalation, and desistance (4).

Studies exploring the mechanisms behind substance misuse typically concentrate on adults, or they present mixed findings for both minors and adults, seemingly assuming that the causes of early-onset substance misuse are alike in both groups. (5–8). However, early initiators may represent a distinct group, necessitating the study of their specific risk factors and pathways (9). They frequently show riskier usage patterns (10), possess distinct risk factors, such as poor parental monitoring or social and family conflicts (10–12), exhibit distinct neural activation patterns (13), experience greater psychosocial issues, and are at a higher risk of developing a severe substance use disorder (14). Adolescence is a developmental period characterized by enormous, simultaneous, and sometimes contradictory influences from family and peer groups. Not only do personal experiences of abuse or neglect increase the risk of substance misuse, but so do those affecting peers (15). Moreover, household dysfunction, including parental substance use disorders (SUDs), heightens the risk for adolescents, particularly in the presence of childhood trauma (16–19).

The adolescent brain is still developing, particularly in areas related to decision-making, impulse control, and emotional regulation, making it more vulnerable to the effects of substances and more susceptible to addictive behaviors (20). Understanding the unique developmental aspects and environmental influences on adolescents is essential for creating effective prevention and intervention strategies tailored to this age group.

Despite the recognized importance of these factors, few studies focus specifically on the complex mechanisms relating adversity to early-onset substance use. Therefore, the purpose of this review is to map the existing literature on the biological and psychosocial pathways leading from adversity to early-onset substance use.

## Methods

The study design is a scoping review, following the PRISMA-ScR (Preferred Reporting Items for Systematic Reviews and Meta-Analyses Extension for Scoping Reviews) checklist and recommendations (21). The pre-specified protocol was uploaded to the Open Science Framework (OSF; 9^th^ Dec 2023; osf.io/z9naq).

Scoping reviews are especially helpful when existing literature has not been thoroughly reviewed or when the literature is complex and varied, making a detailed systematic review difficult. These reviews can be conducted to assess the potential value and scope of a full systematic review. Additionally, scoping reviews can serve as standalone exercises to summarize and share research findings, identify gaps in the research, and provide recommendations for future studies (22).

Unlike systematic reviews that emphasize implications or recommendations for practice, scoping reviews are not intended to support clinical practice decisions. Therefore, assessing the methodological quality or risk of bias of included studies—crucial for reporting effect size estimates—is not a required step and often does not take place (23).

The literature searches were defined as follows: MEDLINE 1946 to December 2023 (OVID), Embase (OVID) 1988 to December 2023, PsycInfo 1806 to December 2023 (OVID), and Web of Science databases were searched individually in December 2023. The MEDLINE (OVID) search strategy was divided into basic and advanced.
Basic: (child* maltreatment OR child* abuse OR child* neglect OR child* adversity OR ACE) AND (substance OR alcohol OR tobacco) AND (mechanism OR pathway OR mediator) AND early onset {Including related terms: 1170 results.
(*) ACE: Adverse Childhood Experience.
Advanced: limit 3 to (“review articles” and humans and (“all child (0 to 18 years)” or “child (6 to 12 years)” or “adolescent (13 to 18 years)”: 42 results.

Three concepts (maltreatment and/or abuse and/or adversity; early-onset substance use, particularly those that are the most frequent initiators to substance use, like alcohol, tobacco, and cannabis; and mediating pathways or mechanisms were combined with Boolean classifier ‘AND.’ For each concept, a broad set of keywords and MeSH terms, combined with the Boolean classifier ‘OR’, was used to identify all existing records. There were no restrictions on language.

In addition to the previous data, grey literature was considered. Google Scholar was searched for additional records. One search was conducted with the following terms: “child maltreatment abuse adversity substance alcohol tobacco cannabis early onset mechanism pathway mediator.” The first 100 results were saved and screened against the eligibility criteria.

### Inclusion criteria

Types of studies: as the explored area is very extensive, only review articles were included. Scoping reviews typically encompass a range of study designs, although this is not prescriptive (22). Every selected review will be analyzed to detect overlapping studies.

Condition or domain being studied: Biological or psychosocial pathways that lead from childhood adversity, abuse, or neglect to early-onset substance use, including tobacco and alcohol.

Participants/population: Substance users aged 17 years or younger with an antecedent of abuse or adversity.

Outcome: any substance use before age 18, including alcohol, tobacco, or illegal substances. Use, abuse, dependence, acute intoxication, or any type of substance use disorder were included.

### Exclusion criteria

There was no restriction by country, language, or publication date.

Types of studies: designs different from review studies were excluded.

Condition of domain being studied: Studies about prevention, diagnosis, treatment, prognosis, comorbidity, or mere association with risk factors that do not consider the psychosocial or biological etiologic mechanism or pathway. Studies about any health outcome that does not include early-onset substance use. Studies about antecedents that differ from childhood exposure to abuse, neglect, or adversity.

Participants/population: Minors without a known antecedent of abuse of adversity. Non-humans. Adults (18+). Studies with mixed results from adults and minors were excluded unless the results for minors could be analyzed separately.

Outcome: Behavioral addictive behaviors.

### Data selection and charting

**Figure 1:**
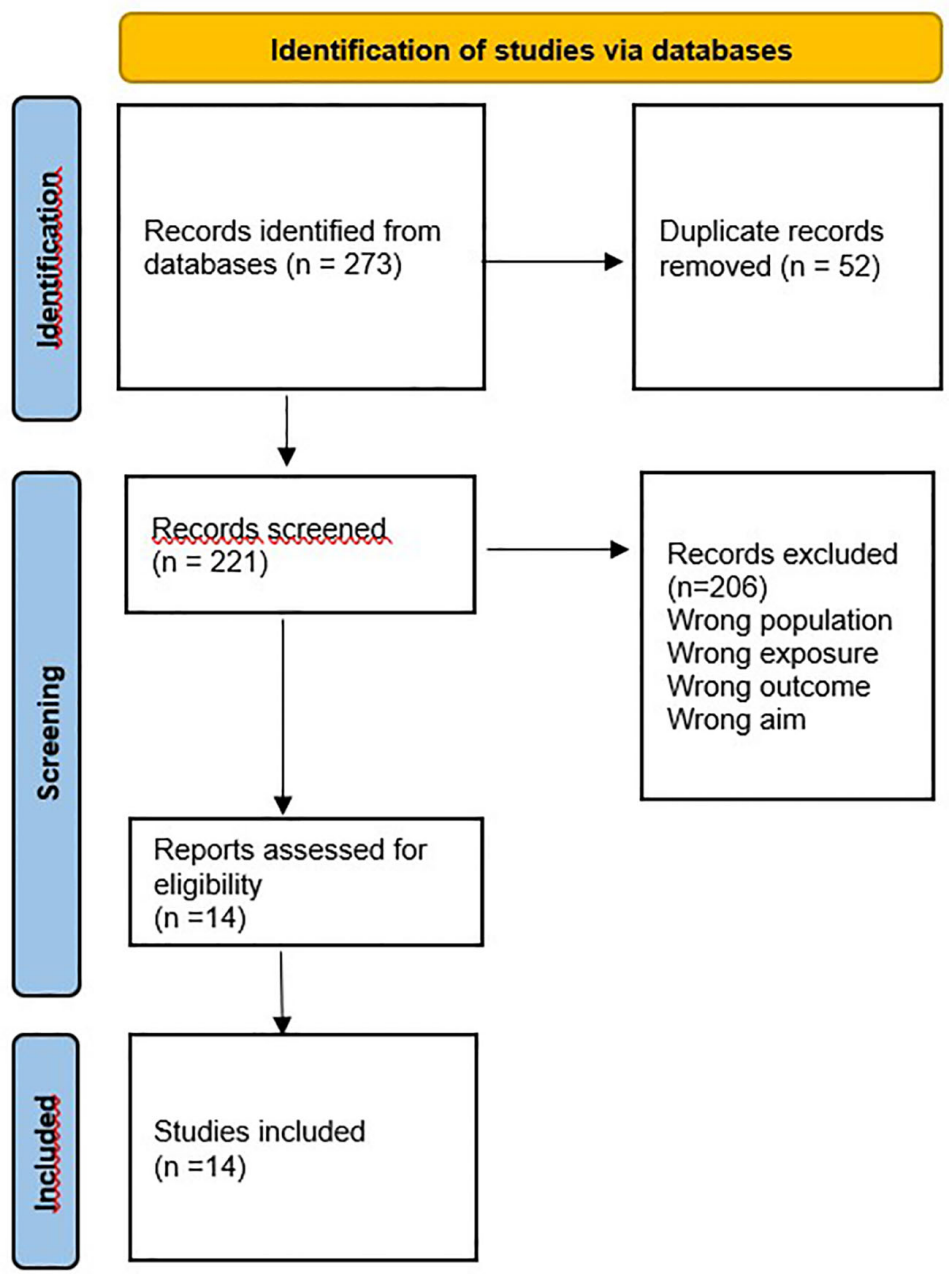
PRISMA flow diagram. From: Page MJ, McKenzie JE, Bossuyt PM, Boutron I, Hoffmann TC, Mulrow CD, et al. The PRISMA 2020 statement: an updated guideline for reporting systematic reviews. BMJ 2021;372:n71. doi: 10.1136/bmj.n71. For more information, visit: http://www.prisma-statement.org/.

The studies gathered from each search were uploaded to Rayyan. All duplicates were removed. All titles and abstracts of the remaining studies were independently screened against the eligibility criteria by two reviewers (MR and AU). Disagreements regarding the inclusion of records were resolved through discussion. Reviews that included studies about substance use outcomes in both adults and minors were thoroughly examined, and only results that applied to children and adolescents below age 18 were charted (MR, AU, AG-P) (see **Figure 1**).

## Results

14 studies were included in this scoping review. A summary of each article is provided in **Table 1**, consisting of relevant data extracted for each included study. The publication dates ranged from 2008 to 2023.

**Table 1:** Overview of evidence by type of exposure, substance used and proposed mechanism.

Author(s)	Exposure	Substance	Proposed mechanism
Acheson (24)	Family histories of alcohol or other SUDs; specific phenotypic characteristics	Any	Increased discounting of delayed reward
Andersen and Teicher (25)	Childhood adversity: abuse, parental loss, witnessing domestic violence or household dysfunction	Any	Highly reactive HPA axis Brain development damage Maturational processes during adolescence
Barker et al. (26)	Environmental exposures, including prenatal and postnatal adversities	Cannabis, alcohol and nicotine	DNA methylation
Cicchetti and Handley (27)	Childhood maltreatment	Any	Externalizing and internalizing pathways
Edalati et al. (2)	Growing up in an unfavorable environment	Any	Stress induction of a negative view of self and others and damage to key brain structures via HPA axis activation and GC production Heightened sensitivity to stress later in life
Etami et al. (28)	Traumatic events	Any	Substances are used to cope with PTSD Drug misuse perpetuates PTSD
Grummitt et al. (5)	Childhood adversity	Any	Individual level moderators Individual level mediators Interpersonal level moderators Individual level mediators Community factors
Hovdestad et al. (29)	Childhood maltreatment	Any	Distress caused by PTSD leads to SU Low self-esteem leads to depression and SU Negative relational strategies
Hoffmann & Jones (30)	Cumulative stressors	Any	Genetic factors related to cortisol regulation, serotonin transport, or DNA methylation Intrapersonal factors such as low self-control, novelty seeking, depressive symptoms, and aggressive tendencies Interpersonal factors such as peer SU
Iacono et al. (31)	Environmental risks: parent-child relations, peer affiliation, stressful and traumatic events and neighborhood effects	Any	A heritable variant of the MAOA enzyme may be implicated in externalizing traits
Lijffijt et al. (32)	Childhood trauma and post-chronic/childhood repeated stress	Any	Stress-induced traits induce initiation Cross-sensitization of reward systems induces progression to regular use Intensified sensitization of reward systems, allostatic changes in stress systems, and increased activity of noradrenergic systems lead to heavy use, dependence, and risk for relapse
Rahim and Patton (33)	Early adversity	Any	Shame and lack of peer acceptance
Sharif-Razi (34)	Early traumatic experiences	Alcohol	Externalizing and internalizing behaviors are mediators The female gender is a moderator
Scheier and Shigeto (35)	Early family risk	Any	A downstream effect caused by adverse child-rearing conditions; intrapersonal factors such as impulsivity, self-regulation, self-esteem, social competence, internalizing and externalizing behaviors; and interpersonal factors such as affiliating with SU-peers

SUD, substance use disorder; HPA, hypothalamic-pituitary-adrenal; GC, glucocorticoid; PTSD, post-traumatic stress disorder; SU, substance use; MAOA, Monoamine oxidase A.

Acheson (24), This study explores how early-life adversity impacts the development of essential neural circuits involved in cognition and emotion regulation, as well as its effects on delay discounting, response inhibition, sensation seeking, and urgency. These processes are associated with a higher likelihood of substance use issues among youth. Delay discounting refers to the tendency to value immediate rewards more highly than those that are delayed. Although some level of delay discounting is common, research shows a link between problematic substance use and an increased tendency to undervalue delayed rewards. Response inhibition refers to the ability to control impulsive reactions, while sensation seeking is characterized by a propensity for engaging in exhilarating and high-risk activities. Urgency, on the other hand, describes the impulsive behavior that arises from intense emotional experiences.

Andersen and Teicher (25). It is proposed that early exposure to life stress may predispose individuals to drug abuse, influenced by three interconnected factors: 1) a highly reactive hypothalamic-pituitary-adrenal (HPA) axis; 2) damage to brain development, especially in the hippocampus, dopamine system, and prefrontal cortex; and 3) maturation processes during adolescence. The study indicates that drug abuse is a developmental disorder characterized by vulnerable periods when exposure to drugs is more likely to result in abuse and dependence. A dysregulated HPA axis can lead to compulsive substance use, while stress-related dysfunctions in the dopamine system, coupled with resulting anhedonia, may heighten the risk of use and addiction. In the absence of the typical mechanisms that curb substance use, typically found in the hippocampus and prefrontal cortex, drug-seeking behaviors tend to escalate. The impact of early adversities on brain development is often delayed in showing effects but may suddenly surface as the prefrontal cortex matures during adolescence.

Barker et al. (26) evaluated the influence of DNA methylation (DNAm) as a potential mediator between environmental risks and psychopathological outcomes, including substance use in adolescents. Preliminary evidence indicates that environmental factors can modify DNAm in offspring; however, limited research has focused on how DNAm may impact cannabis, alcohol, and nicotine use in children and adolescents. The function of DNAm as a mediator in this context remains unclear due to the limited number of available studies.

Cicchetti and Handley (27). Research reveals an externalizing pathway to substance use disorder. Childhood behaviors like disinhibition, aggression, and rule-breaking are significant indicators. While general parenting practices can help reduce substance use in teenagers, peer associations with substance users strongly predict adolescent substance use. Maladaptive parenting strategies often interact with a child’s temperament and peer relationships, increasing this risk. Moreover, the family environment may mirror a pattern of parental substance use disorders. Studies indicate that childhood experiences, such as physical abuse, conduct problems, parental drug use, and friendships with substance-using peers, independently forecast the likelihood of drug disorders in late adolescence. Evidence also points to an internalizing pathway, where positive views of substance use as a means of relieving distress correlate with problematic consumption. However, research presents mixed findings. Some studies suggest that changes in the hypothalamic-pituitary-adrenal (HPA) axis response to stress may represent another element of these internalizing pathways. It is hypothesized that early life adversity and substance use disorder (SUD) might be influenced by a highly reactive HPA axis, prompting individuals to resort to substances as a coping mechanism for negative emotions.

Edalati et al. (2) proposed a dual-process model that links the risk of growing up in harmful environments to neurobiological vulnerabilities. The early development of subcortical circuitry, alongside the slower maturation of frontal cortical circuitry, may lead to heightened reward-seeking behaviors, particularly in the absence of sufficient behavioral control, thus making adolescents more prone to risk-taking. Moreover, these neural developments render the brain more susceptible to the immediate effects of drugs, placing adolescents who begin using substances early at a greater risk for heavy or uncontrolled use and making them more sensitive to the addictive properties of these substances. Adverse childhood experiences (such as violence, neglect, abuse, and household dysfunction) can foster a negative self-image and perception of others. Experiencing such adverse events during critical developmental periods can lead to lasting changes in crucial brain structures and functions, thereby adversely affecting the brain’s response to stress. Early stress activates the hypothalamic-pituitary-adrenal axis, leading to increased glucocorticoids (GCs) that aim to lower the activity of these systems. Elevated GC production due to early stress can have permanent effects on the brain areas that regulate the release of these hormones. These mechanisms impact various brain regions based on their developmental stage, receptor density for GCs, and stress sensitivity. The brain areas most affected by early stress and adversity include the amygdala, hippocampus, cerebellum, corpus callosum, and prefrontal cortex (PFC). Neuroimaging studies of children with adverse childhood experiences show that disruptions in normal brain development can lead to a chain reaction resulting in neurocognitive deficits in memory, reward processing, intellectual abilities, and self-control. Specifically, exposure to traumatic experiences and violence alters the PFC’s normal development and its functions, including inhibitory control, abstract reasoning, problem-solving, planning, and related personality traits such as impulsivity and sensation-seeking, which increase the likelihood of substance misuse and developing a substance use disorder (SUD). Additionally, adverse childhood experiences can lead to dysfunctional memory associations that tie the self-concept to maladaptive beliefs, including self-blame, negative thoughts about oneself, and low self-esteem. This maladaptive self-image, coupled with difficulties in behavioral inhibition and reward processing, places individuals who have faced maltreatment at high risk for SUD. The impact of childhood adversity also correlates with a greater sensitivity to stress and predicts further adverse experiences in elementary and middle school students, heightening the likelihood of using substances as a means to cope with stress and negative emotions from harsh environments. Similarly, bullying significantly contributes to a negative self-image and low self-esteem during adolescence. As a traumatic event, bullying increases the risk of adverse outcomes, including substance use among adolescents.

Etami et al. (28) suggest that trauma and substance misuse are linked, as adolescents might turn to drug abuse as a coping mechanism for PTSD. Such drug misuse can result in neuroadaptations affecting learning processes, which in turn aid in the consolidation of traumatic memories. This indicates a shared engagement of neurocircuitry triggered by stress and drug misuse, particularly involving alterations in limbic brain areas.

Grummit et al. (5) conducted a systematic review of the literature, identifying mediators and moderators in the relationship between childhood adversity and substance use outcomes. Individual-level mediators exhibited the most reliable effect sizes, particularly externalizing behavior and substance use coping motives. Additionally, among individual-level moderators, factors such as religiosity and depressive symptoms weakened this relationship. At the interpersonal level, peer and mother-child relationships acted as mediators between adversity and substance use, with relationship quality and family cohesion serving as moderators. Community factors were infrequently analyzed; school mobility and educational achievement mediated 14% and 28% of the overall impact of childhood adversity on substance use.

Hovdestad et al. (29) examined the processes that account for heightened vulnerability to substance use among adolescents following early negative experiences. They propose three explanatory frameworks: the Post-Traumatic Stress Disorder (PTSD) framework, which posits that distress triggers substance use; the Self-Dysfunction framework, in which low self-esteem contributes to depression and subsequent substance use; and the Relationship Difficulty framework, which suggests that insecure attachments or challenging relationships with parents result in isolation or conflict with peers.

Hoffmann and Jones (30) highlight the consistent yet subtle link between stressors and adolescent substance use. Research suggests that this relationship is influenced by genetic factors related to cortisol regulation, serotonin transport, and DNA methylation, as well as personal factors such as peer substance use.

Iacono et al. (31) provide evidence that supports the existence of a significantly heritable latent externalizing trait; however, empirical evidence regarding its genetic mechanisms is lacking. Variants in the gene responsible for the Monoamine Oxidase A (MAOA) enzyme may be linked to externalizing traits.

Lijffijt et al. (32) reviewed literature about how stress influences the course of addiction. The initiation of substance use may be linked to stress-induced characteristics, such as heightened stress reactivity and increased impulsivity. There appears to be a connection between the escalation to abuse and stress-related cross-sensitization of reward systems, which enhances the motivational impact of lower substance doses, leading to an earlier transition to regular use and higher dosages. This mechanism likely works in conjunction with the previously mentioned trait-like factors. Sensitization may be influenced by the use of substances, with individuals exhibiting high trait impulsivity being especially vulnerable to sensitization. Ultimately, the shift to dependence and the risk of relapse could be connected to both substance- and stress-induced sensitization of reward pathways, allostatic alterations in stress systems, their links to amygdala-driven negative reinforcement, and heightened sensitivity or reactivity of noradrenergic systems.

Rahim and Patton (33) examined how shame-proneness relates to negative outcomes, including diminished functioning, increased psychopathology, and early substance use. They proposed that adolescents who are shame-prone, stemming from early adversity, tend to view themselves unfavorably in comparison to their peers and may resort to coping mechanisms like criminal activities or risk-taking behavior to seek acceptance from friends.

Sharif-Razi (34) discovered a notable positive correlation between early traumatic experiences and problematic alcohol use among adolescents. This correlation was more pronounced in females, with gender acting as a moderator and both externalizing and internalizing behaviors serving as mediators.

Scheier and Shigeto (35) examined the studies on developmental cascade models, exploring the causal connections between substance use and early family risk. The researchers found limited evidence linking early parental socialization to substance use etiology, along with methodological concerns. Parental substance abuse led to a broader range of negative child-rearing conditions. Nevertheless, even in these situations, the journey toward substance use was still influenced by friendships with peers who use substances. Several additional factors also played a role, including intrapersonal and interpersonal elements such as impulsivity, self-regulation, self-esteem, social competence, and both internalizing and externalizing behaviors.

## Discussion

The pathways from early adverse experiences and early-onset substance use are complex and multidimensional. Early experiences have a cascading effect on subsequent stages, influencing both neurobiological development and the types of experiences the child will be exposed to (36).

The exposed data found that it is crucial to analyze the pathways or mechanisms that lead from childhood trauma to early-onset substance use without assuming they are the same as those in adults. It is vital for several reasons, including developmental differences, unique risk factors, long-term outcomes, and biopsychosocial factors.

This scoping review finds that most research emphasizes psychosocial mechanisms, supporting the self-medication hypothesis, particularly at the onset of substance use. Adolescents may use substances as a maladaptive coping strategy when experiencing shame, internalizing or externalizing symptoms, and post-traumatic stress. As substance use becomes chronic, the motivation may shift from positive reinforcement to negative reinforcement due to changes in cognition, stress response, and the reward system.

However, while the self-medication hypothesis remains accepted (37, 38), trauma-exposed youths also develop other nonsubstance addictive behaviors, such as internet or social media addiction (39, 40), implying that other mechanisms are likely involved.

Concerning developmental differences, adolescents and adults are at different stages of cognitive, emotional, and social development. The adolescent brain is still developing, particularly in areas such as decision-making, impulse control, and risk assessment (20). For example, trauma can disrupt the development of the stress response system, leading to increased vulnerability to substance use (41, 42). Once initiated, substance use in young people can disrupt critical developmental processes. Adolescence is a peak time for initiating substance use, with tobacco and alcohol typically preceding the use of illicit drugs (43).

Concerning unique risk factors, adolescents are exposed to risk factors that are different from those of adults. For instance, peer pressure, academic stress, and the desire for social acceptance are particularly salient during adolescence (44). Although solitary use may be more characteristically linked to childhood adversity, both solitary and social use are related to trauma (45, 46).

Additionally, adolescents might use substances as a means of coping with developmental challenges specific to this life stage while also considering long-term outcomes. The gateway theory posits that less harmful substance use in adolescence may lead to illicit drugs later in life. Individuals who start using tobacco, alcohol, or marijuana at a young age are more prone to using other substances as adults (40). Adolescents who begin using substances and subsequently develop SUD typically progress through multiple sequential stages: experimental or social use, escalation, maintenance, abuse, and eventual dependence. However, there are subgroups of users who never escalate, maintain non-dependent use for decades, experience intermittent periods of cessation, or achieve permanent abstinence (4, 47, 48). Anyway, the risk associated with substance use during childhood and adolescence extends beyond the potential for developing sustained use or long-term dependence. It also includes the risk of accidents, violence, psychotic disorders, affective and anxiety disorders, self-harm, suicide, infectious diseases such as HIV, overdose, cognitive impairment, school failure, and delinquency (49).

The fourth aspect considered in this review is related to biopsychosocial factors. Developmental outcomes related to substance use are presumed to result from a combination of genetic and environmental protective and risk factors, which have a nonlinear relationship. Some factors can exacerbate or attenuate the effects of others. Environmental and contextual conditions are potentially malleable, while genetic factors are less amenable to change (4). Adolescents might have different biopsychosocial factors influencing their substance use. For instance, research indicates that early substance use may lead to disconnection from parents and abstaining peers, thereby increasing susceptibility to user peer pressure and the risk of progressing to illegal drugs (50, 51). However, the peer effect may be stronger when adolescents are genetically predisposed to substance use (52). Among environmental risk factors, exposure to stressful events in childhood is a well-known factor that increases susceptibility to substance use, escalation, relapse, and treatment resistance (5).

These explanatory models are crucial as they may point to mediators that could be usefully targeted in interventions with adolescents with a childhood maltreatment background (29). For instance, research indicates that the right dorsolateral prefrontal cortex (DLPFC) provides inhibitory control that enhances resilience to substance use. Consequently, interventions designed to boost this neural resilience through cognitive-behavioral techniques may mitigate the development-related psychological imbalance between reward and inhibitory systems while improving executive function and self-regulation (53). Additionally, fostering connections with prosocial peers who do not use drugs, improving relationships with parents, and encouraging meaningful involvement in school or community are shown to be protective factors that mitigate the effects of childhood adversity on substance use (5, 54, 55).

In summary, it is crucial to understand the specific mechanisms that operate in this particularly vulnerable developmental stage. This understanding is essential for developing preventive strategies and treatments to mitigate the damage caused by early adversity.

To our knowledge, this is the first scoping review to investigate biological and psychosocial pathways that lead from childhood adversity to early-onset substance use.

## Limitations

The present review has some limitations. Firstly, while there is consistent evidence reporting positive associations between childhood adversity and substance misuse, studies about the mechanisms that explain this association are scarce, particularly in minors. The results do not include reviews from Asia or Africa, where substance use disorders in minors may be present differently. A second caution is that of causality. The relationship between adversity and substance use is frequently based on associations, so it should not be implied as causal. Additionally, adversity often occurs in environments where other confounding factors can limit the ability to infer causality without a rigorous study design. Further research is needed to understand better the biological and psychological pathways leading to substance use during childhood and adolescence. Ultimately, the concept of adversity is too heterogeneous to yield consistent and comparable results across studies.
